# Intronic Sequence Regulates Sugar-Dependent Expression of *Arabidopsis thaliana Production of Anthocyanin Pigment-1/MYB75*

**DOI:** 10.1371/journal.pone.0156673

**Published:** 2016-06-01

**Authors:** Bettina E. Broeckling, Ruth A. Watson, Blaire Steinwand, Daniel R. Bush

**Affiliations:** Department of Biology, Colorado State University, Fort Collins, Colorado, United States of America; Purdue University, UNITED STATES

## Abstract

Sucrose-specific regulation of gene expression is recognized as an important signaling response, distinct from glucose, which serves to modulate plant growth, metabolism, and physiology. The Arabidopsis MYB transcription factor *Production of Anthocyanin Pigment-1* (*PAP1)* plays a key role in anthocyanin biosynthesis and expression of *PAP1* is known to be regulated by sucrose. Sucrose treatment of *Arabidopsis* seedlings led to a 20-fold induction of *PAP1* transcript, which represented a 6-fold increase over levels in glucose-treated seedlings. The *PAP1* promoter was not sufficient for conferring a sucrose response to a reporter gene and did not correctly report expression of *PAP1* in plants. Although we identified 3 putative sucrose response elements in the *PAP1* gene, none were found to be necessary for this response. Using deletion analysis, we identified a 90 bp sequence within intron 1 of *PAP1* that is necessary for the sucrose response. This sequence was sufficient for conferring a sucrose response to a minimal promoter: luciferase reporter when present in multiple copies upstream of the promoter. This work lays the foundation for dissecting the sucrose signaling pathway of *PAP1* and contributes to understanding the interplay between sucrose signaling, anthocyanin biosynthesis, and stress responses.

## Introduction

Regulation of genes by sugars such as glucose and sucrose has been well documented in plants and plays an important role in plant growth, development, and physiology [1–6]. Although it can be difficult to attribute signaling responses to a specific sugar molecule as opposed to one of its metabolites or catabolites, there is strong evidence for distinct signaling pathways for both glucose and sucrose [7–9]. Compared with glucose, sucrose-specific signaling pathways are not well characterized, and a sucrose-specific sensor functioning similar to that of hexokinase in glucose signaling has not been identified although several have been proposed [3, 7].

Genes regulated specifically by sucrose are generally defined as those that do not respond in a similar fashion to glucose or fructose. The best known examples of sucrose regulated genes are the *Beta vulgaris* sucrose transporter *BvSUT1* from sugar beet [5, 9], patatin from potato [10, 11], and the Production of Anthocyanin Pigmentation-1 *PAP1/MYB75* transcription factor that regulates anthocyanin biosynthesis in *Arabidopsis thaliana* [12, 13]. For these genes, there is minimal regulation in the presence of glucose or fructose. Other genes such as chalcone synthase [14], *rolC* [15], and beta amylase [16, 17] respond to glucose and other sugars yet show a stronger response to sucrose, while for others comparable responses have been observed for both sucrose and glucose [18–20]. It should also be noted that in many cases differences in gene responses to sugars have been reported. For example, glucose and fructose-induced expression of potato proteinase inhibitor II was reported to be equal to or half the response observed with sucrose [21, 22]. And higher expression of *PAP1* in glucose versus sucrose-treated seedlings has also been reported [23]. This is most likely due to differences in how experiments were performed and/or underlying metabolic changes in sugar content and identity.

For some of these genes sugar response elements or sequences important for sugar regulation have been identified and nuclear binding proteins or activities characterized [4]. Genetic approaches have led to identification of genes that play important roles in regulating sugar expression of specific genes [24, 25]. However, overall the molecular pathways involved in sucrose-signaling are not well understood.

We are using *Arabidopsis PAP1*, a known sucrose-regulated gene, as a model to dissect sucrose signaling. Teng *et al* first identified *PAP1* as important for sucrose-induced anthocyanin accumulation in *Arabidopsis* and showed that a *PAP1* knock-out mutant lacked this response [12]. Solfanelli *et al* further showed that expression of *PAP1* was specific to sucrose and not glucose or fructose [13], although a more recent paper reported higher *PAP1* expression in glucose-treated seedlings compared to sucrose-treated seedlings [23]. Sucrose-induced expression of *PAP1* has been shown to be modulated by hormones [23, 26–28], mutations in the *AtSUC1* sucrose transporter [29], and alterations in calcium signaling [30]. In many cases it is not clear if these factors directly impact *PAP1* expression or are secondary due to changes in endogenous sugar content. Although there have been several reports on the expression of *PAP1* in response to sugars, sequences important for the sucrose-responsiveness of *PAP1* have not been identified.

To begin to dissect the sucrose-regulated pathway of *PAP1* we have localized sequences important for this response to an intron and further show that this intronic sequence can confer sucrose-responsiveness to a reporter gene. We also discuss the implications of *PAP1* regulation by sucrose and how this might contribute to regulation of anthocyanin biosynthesis in plants.

## Results

### Sucrose specificity of anthocyanin biosynthesis

**Fig 1:**
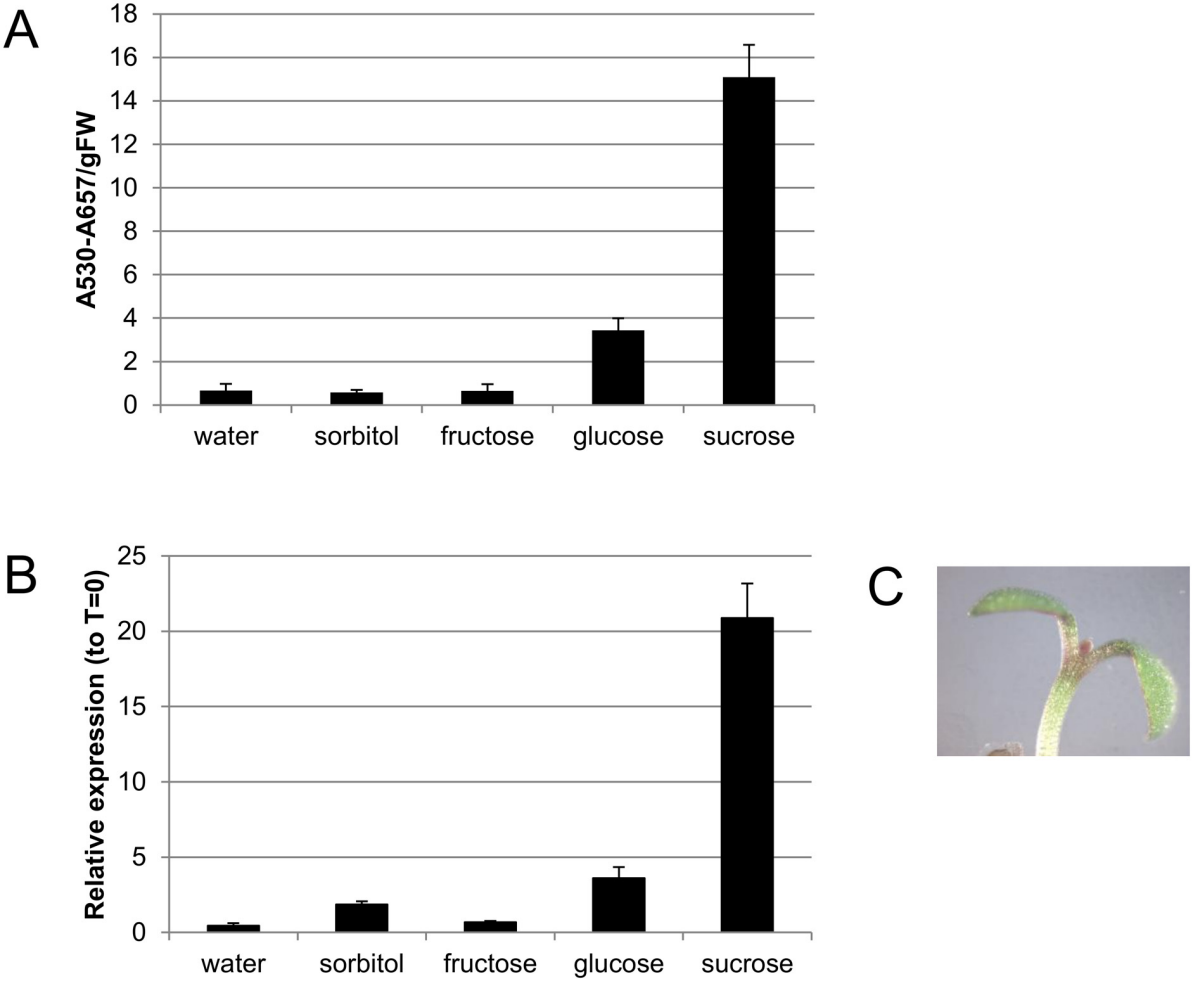
Sucrose-specific anthocyanin biosynthesis in light grown seedlings. A. Anthocyanin quantification in 4 day old seedlings treated with water or 90 mM sugar for 48 hours in continuous light. B. RT-qPCR of *PAP1* transcript in 4 day old seedlings treated with water or 90 mM sugar for 24 hours in continuous light. C. Anthocyanin pigmentation in a seedling treated with 90 mM sucrose for 24 hours.

To confirm the sucrose-specific induction of anthocyanin biosynthesis, *Arabidopsis Col-0* seeds were germinated in one-half-strength liquid Murashige and Skoog (MS) media and seedlings were treated with water or various sugars after 4 days of growth in continuous light. Seedlings were harvested 48 hours later and anthocyanins quantified (Fig 1A). Sucrose treated seedlings accumulated the most anthocyanin, although glucose treated seedlings also accumulated anthocyanin relative to the water treated control. These results are similar to those reported by Teng *et al* [12]. Expression of *PAP1* was examined by RT-qPCR 24 hrs after addition of water or various sugars (Fig 1B). *PAP1* transcript abundance paralleled anthocyanin accumulation; *PAP1* levels were 20- and 3.6-fold greater after sucrose and glucose treatment, respectively.

### Identification of sequences important in conferring sucrose responsiveness of *PAP1*

To define sequences important in governing the sucrose-induced expression of *PAP1*, we generated *PAP1* promoter: reporter fusions with β-galactosidase and luciferase (*PAP1pro*:*GUS* or *LUC*). The promoter sequence was defined as 2006 bp upstream of the *PAP1* start codon. A known sucrose response element (SRE), SURE-2, was identified in the promoter of *PAP1* using the Plant cis-acting regulatory DNA element (PLACE) database [31]. SURE-1 (TTTTCTATT) and SURE-2 (AATACTAAT) elements were initially identified as important in conferring sucrose responsiveness of patatin, a lipolytic acyl hydrolase from potato [32]. SURE-1 and SURE-2 share sequence similarity with one another (5 out of 9 identical nucleotides) and with SP8 elements [32, 33]. The similarity between the SURE-2 elements in the *PAP1* and patatin promoters extended beyond the 9 bp core element to 17 bp with only one discrepancy (patatin sequence: TATATAATACTAATAAA; *PAP1* sequence has a T in place of the underlined A).

**Fig 2:**
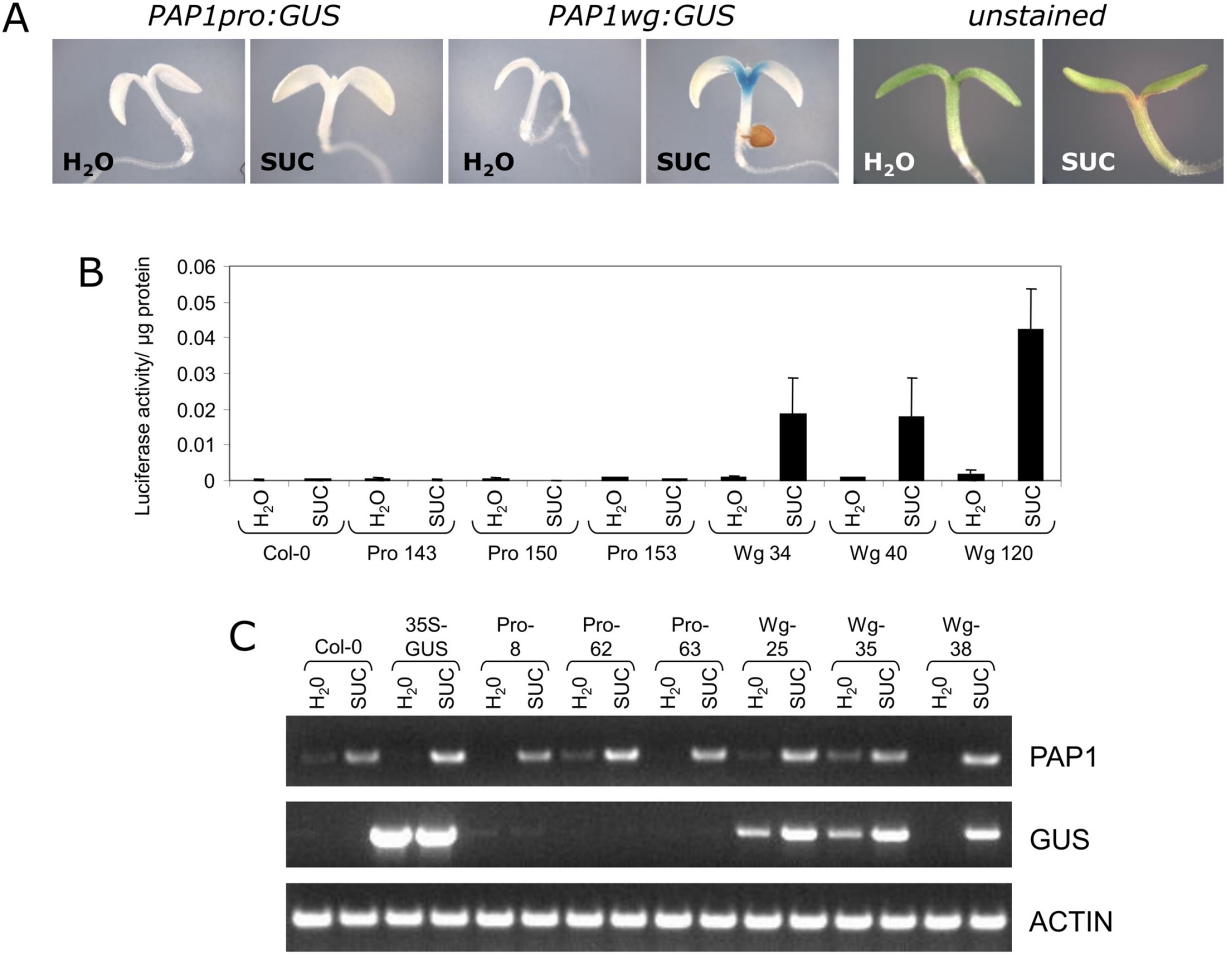
Sucrose-responsiveness of *PAP1* promoter and whole gene constructs. A. Representative GUS staining of 4 day old seedlings treated with water or 90 mM sucrose for 24 hours. In sucrose-treated seedlings, anthocyanin pigmentation is observed at the base of the cotyledons (unstained seedlings). B. Luciferase activity of *PAP1* promoter and whole gene constructs in 4 day old seedlings treated with water or 90 mM sucrose for 24 hours. C. RT-PCR of *PAP1*, *GUS*, and actin transcripts in 4 day old *PAP1pro*:*GUS* and *PAP1wg*:*GUS* seedlings treated with 90 mM sucrose for 4 hours. Numbers following Pro and Wg abbreviations refer to independent transgenic lines.

Constructs were transformed into *Col-0* and transgenic plants were generated. For these and all subsequent experiments, 5–10 single copy homozygous lines were generated per construct and data is shown for 2–4 representative lines per construct. When plants were tested for sucrose-induced GUS or LUC activity as described above, no activity was observed (Fig 2A and 2B). RT-PCR of sucrose-treated *PAP1pro*:*GUS* plants showed that even though *PAP1* transcript was present, the *GUS* transcript was not expressed in response to sucrose (Fig 2C).

**Fig 3:**
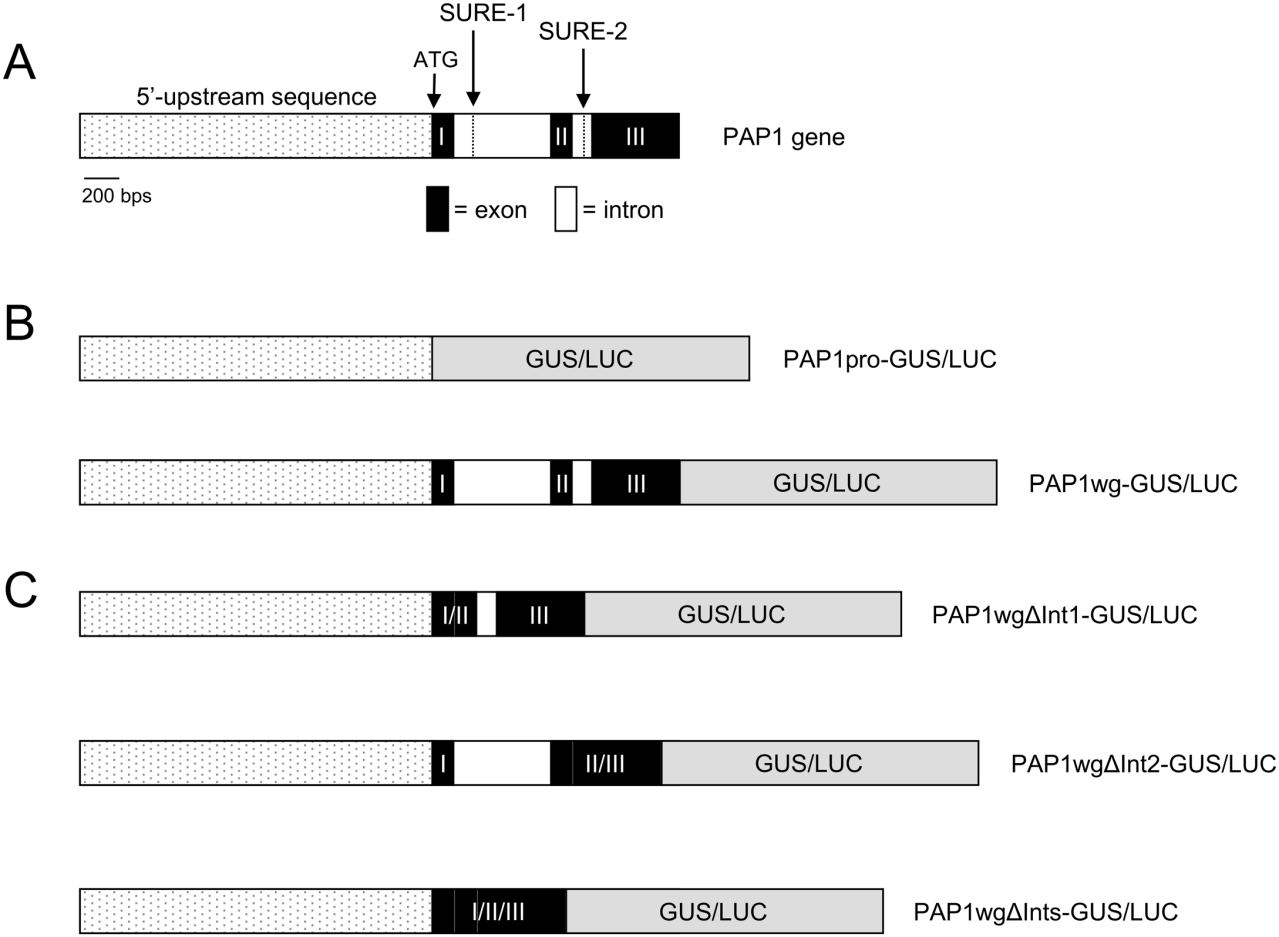
*PAP1* gene organization and reporter gene constructs. A. Schematic of *PAP1* gene showing the location of the SURE-1 and SURE-2 elements. B. Schematic of *PAP1* reporter gene constructs.

Several genes, including sucrose transporters, have been shown to require additional sequences contained within exons and introns for proper gene expression [34]. To test whether sequences in the exons and introns are important for sucrose-induced expression of *PAP1*, we fused the *PAP1* promoter plus all exons and introns to *GUS* or *LUC* (*PAP1wg*:*GUS* or *LUC*) (Fig 3B). Transgenic plants harboring these constructs were generated and tested for GUS and LUC activity. Both reporter constructs showed sucrose-dependent reporter gene activity (Fig 2A and 2B). Furthermore, the *PAP1wg*:*GUS* lines had GUS staining at the base of the cotyledon and the upper part of the hypocotyl, reminiscent of anthocyanin pigmentation in seedlings (Figs 1C and 2A). RT-PCR of these plants showed that the *GUS* transcript was expressed in response to sucrose, similar to the expression of the endogenous *PAP1* transcript (Fig 2C).

### *PAP1* gene expression during plant development

To determine whether *PAP1pro*:*GUS* plants correctly reported *PAP1* expression at other developmental stages we compared the pattern of GUS expression of *PAP1pro*:*GUS* and *PAP1wg*:*GUS* lines. Seeds were germinated on MS media plus 2% sucrose and plants were harvested at various time points. In *PAP1wg*:*GUS* lines GUS staining was observed in the hypocotyl at 3, 5, and 7 days; no GUS staining was observed in *PAP1pro*:*GUS* lines (S1 Fig). Fourteen day old *PAP1wg*:*GUS* plants had strong GUS staining in leaves and cotyledons and faint patches of staining could also be observed in the roots (S2 Fig). No GUS staining was observed in *PAP1pro*:*GUS* lines. At 20 days *PAP1wg*:*GUS* lines had GUS staining in the leaves, mostly along the major veins and petioles (S3 Fig). *PAP1pro*:*GUS* lines did not show GUS staining except for a very small spot on the primary root several mm from the base of the rosette. This was distinct from the pattern of GUS staining observed in *PAP1wg*:*GUS* lines, in which GUS staining was limited to the top of the primary root near the base of the rosette. RT-PCR of 20 day old plants indicated *PAP1* was expressed in both shoots and roots, while *PAP1pro*:*GUS* lines only showed GUS transcript in the roots (S3 Fig).

**Fig 4:**
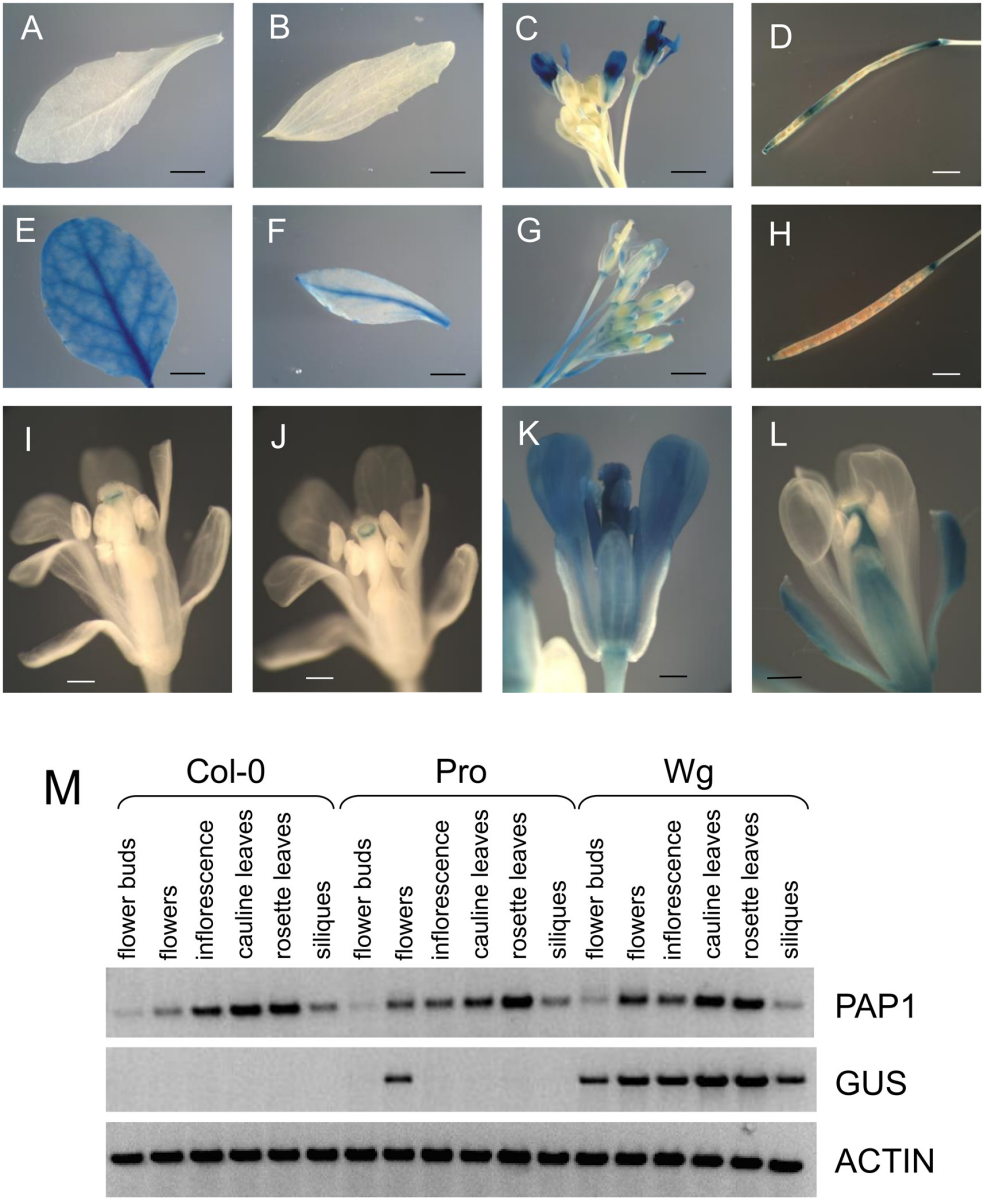
GUS expression patterns (A-L) and RT-PCR (M) of *PAP1pro*:*GUS* (A-D, I-K) and *PAP1wg*:*GUS* (E-H, L) lines. *PAP1pro*:*GUS* lines did not show expression in rosette (A) or cauline (B) leaves. Siliques usually showed patchy staining (D). No staining was observed in flower buds (C). Immature flowers (I, J) showed a band of staining just under the stigma, while mature flowers (C, K) showed expression in flower petals, ovaries, and filaments. *PAP1wg*:*GUS* lines showed staining in rosette (E) and cauline (F) leaves, flowers at all stages (G), and siliques (H). GUS staining was observed in sepals, ovaries, and filaments of flowers (L). Siliques of *PAP1pro*:*GUS* plants were not uniformly or consistently stained in this experiment. M. RT-PCR of 5 week old plants expressing *PAP1* promoter and whole gene constructs. Scale bars are 3 mm in A, B, E, and F, 2 mm in C, D, G, and H, and 0.5 mm in I-L.

In flowering plants (28–42 days old) there was some variation in the pattern and intensity of GUS staining in different experiments that may be due to differences in plant development or environmental conditions. In all plants examined, *PAP1pro*:*GUS* lines showed GUS staining in petals, ovaries, and filaments of mature flowers and in some experiments staining was also observed in stigma and anthers (Fig 4C and 4K). Interestingly, in *PAP1pro*:*GUS* lines flower buds had little to no staining and immature flowers only showed a discrete band of staining just under the stigma, suggesting that the observed GUS expression pattern may be under developmental control (Fig 4C, 4I and 4J). Siliques were usually stained, but seeds were not (Fig 4D). In some experiments GUS staining could also be observed in cauline leaves (weak staining along the major vein), secondary inflorescences, and pedicels close to the junction with flowers and siliques. No staining was observed in rosette leaves (Fig 4A), stem, primary inflorescences, and sepals (Fig 4K).

In comparison, GUS staining of *PAP1wg*:*GUS* lines was more uniformly distributed and present in most organs including rosette and cauline leaves (most notably along veins), flowers, and pedicels (Fig 4E–4G). In contrast to the staining observed in *PAP1pro*:*GUS* lines, *PAP1wg*:*GUS* lines showed GUS staining in sepals, but not petals (Fig 4L). The lack of GUS staining in petals is consistent with the lack of anthocyanins in *Arabidopsis* petals [35]. GUS staining was also observed in ovaries and filaments in flowers, but not stigma or anthers (Fig 4L). In some experiments staining was observed in stem and primary and secondary inflorescences, and siliques (Fig 4H). No GUS staining was observed in seeds.

GUS staining and *GUS* transcript observed in *PAP1wg*:*GUS* lines correlated well with native *PAP1* transcript as detected by RT-PCR, which showed *PAP1* transcript in rosette and cauline leaves, inflorescence, flower buds, flowers and siliques (Fig 4M). *PAP1pro*:*GUS* lines only showed GUS transcript in flowers, but not in any other tissues. These results suggest that the *PAP1* promoter is not correctly reporting *PAP1* expression in *Arabidopsis*. Our expression data for *PAP1wg*:*GUS* lines correlates well with expression data in the AtGenExpress atlas of *Arabidopsis* development [36]. In this dataset expression values of *PAP1* are fairly low (with the exception of senescing leaves), but expression is detected in rosette and cauline leaves, 7 day old seedlings, and stage 15 flowers (including petals, stamen, and carpels). Taken together, the whole gene construct is not only necessary for the response to sucrose as it contains sequences necessary for controlling *PAP1* expression as a function of tissue and development.

The expression pattern of a similar *PAP1pro*:*GUS* construct has been reported [37]. GUS expression was reported in hypocotyls and cotyledons of 2–3 day old seedlings and young emerging leaf tissue/primordia of 5–7 day old seedlings. As discussed above, we did not observe any GUS staining in seedlings of our *PAP1pro*:*GUS* lines when grown under our conditions or those reported by Gonzalez et al.

### Importance of intronic sequences in conferring sucrose responsiveness of *PAP1*

**Fig 5:**
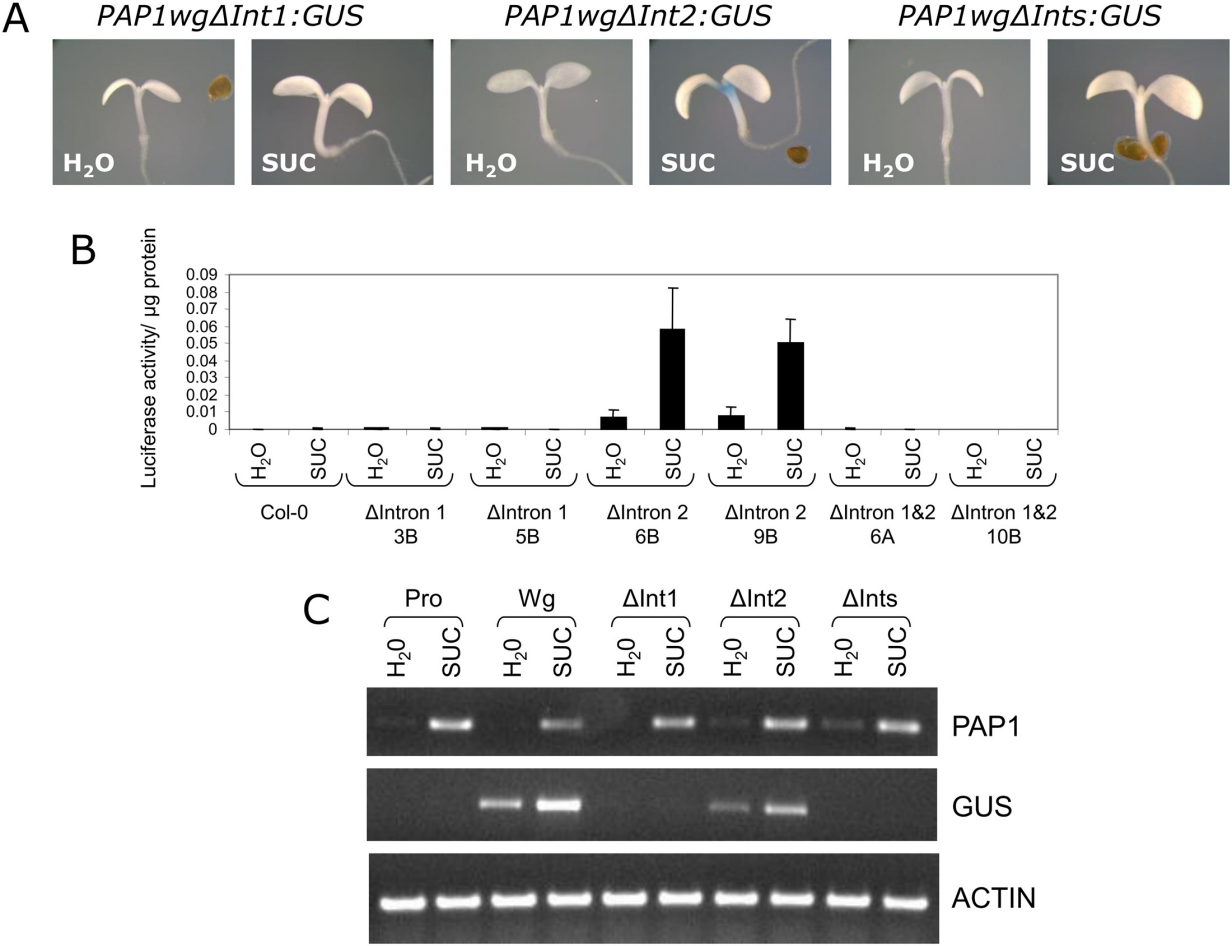
Sucrose-responsiveness of *PAP1* delta intron gene constructs. A. Representative GUS staining of 4 day old seedlings treated with water or 90 mM sucrose for 24 hours. B. Luciferase activity of *PAP1* delta intron constructs in 4 day old seedlings treated with water or 90 mM sucrose for 24 hours. C. RT-PCR of *PAP1*, *GUS*, and actin transcripts in 4 day old seedlings treated with 90 mM sucrose for 4 hours.

To further define sequences important in controlling sucrose-dependent expression of *PAP1*, we generated additional reporter gene constructs lacking one or both introns of *PAP1* (*PAP1wgΔInt1*:*GUS* or *LUC*, *PAP1wgΔInt2*:*GUS* or *LUC*, *PAP1wgΔInts*:*GUS* or *LUC*) (Fig 3C). Transgenic plants were generated and tested for reporter gene activity. Constructs lacking intron 1 or both intron 1 and 2 (*PAP1wgΔInt1*:*GUS* or *LUC* and *PAP1wgΔInts*:*GUS* or *LUC*) did not have reporter gene activity in response to sucrose (Fig 5A and 5B). This was confirmed by RT-PCR, which showed no *GUS* transcript expressed in response to sucrose (Fig 5C). However, the construct lacking intron 2 (*PAP1wgΔInt2*:*GUS*) retained sucrose-induced reporter gene activity and *GUS* expression in response to sucrose. The pattern of GUS staining observed for *PAP1wgΔInt2*:*GUS* in seedlings and plants was identical to that observed for the construct containing the entire *PAP1* gene (*PAP1wg*:*GUS*) (Figs 2A & 5A). However, the pattern of GUS staining in *PAP1wgΔInt1*:*GUS* and *PAP1wgΔInts*:*GUS* plants was much more limited. In these plants staining was observed in the major vein of rosette leaves with more intense staining near the base of the rosette and weaker staining towards the tip of the leaves (S4 Fig). Staining was also observed at the base of cauline leaves at the attachment point to the inflorescence. No other staining was observed in these lines.

### Role of SURE-1 in the sucrose-response

**Fig 6:**
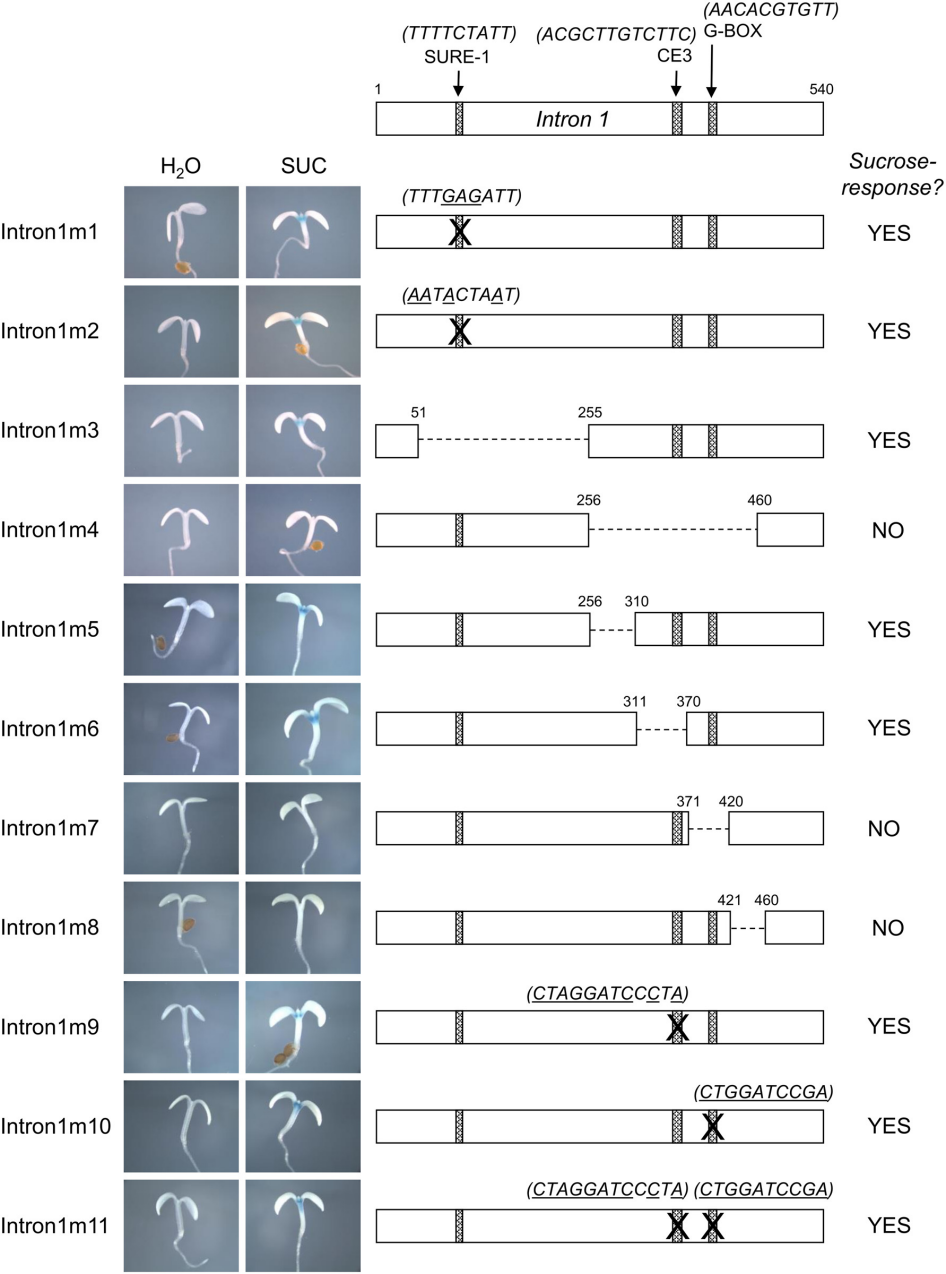
Summary of sucrose responsiveness of *PAP1* Intron 1 mutants. Detailed schematic depicting mutations of *PAP1* intron 1 and representative GUS staining of 4 day old seedlings treated with water or 90 mM sucrose for each mutation.

Having identified intron 1 as important for the sucrose response of *PAP1*, we next used PLACE to scan intron 1 for known cis-elements and identified the SURE-1 sucrose response element (TTTTCTATT) (Fig 3A). Interestingly, we also identified another SURE-2 element (AATACTAAT) within intron 2, which was shown to be dispensable for the sucrose response. To determine whether the SURE-1 element in intron 1 was necessary for the sucrose-responsiveness of *PAP1*, we generated 2 constructs in which the SURE-1 element was directly mutagenized. For intron1m1, the SURE-1 element (TTTTCTATT) was mutated to TTTGAGATT according to Grierson *et al*, who showed that this mutation abolished binding of a protein from nuclear extracts of potato tubers in electrophoresis mobility shift assays [32]. In the second construct, intron1m2, 4 nucleotides of the SURE-1 element were mutagenized to convert the SURE-1 element (TTTTCTATT) to the SURE-2 element (AATACTAAT). The SURE-2 element is present in the promoter and intron 2 of *PAP1* and does not appear to be essential for the sucrose response as described above. The mutations were incorporated into *PAP1wg*:*GUS* constructs and neither mutation was observed to alter splicing. Both mutants retained sucrose-induced GUS activity and *GUS* expression in response to sucrose (Fig 6). This suggests that the SURE-1 element in intron 1 is not important in conferring sucrose responsiveness to *PAP1*.

### Deletion analysis defines a 90 bp region in intron 1 as important for the sucrose-response of *PAP1*

In order to identify sequences in intron 1 that are important for the sucrose-response of *PAP1* we deleted regions of intron 1 within the context of the *PAP1wg*:*GUS* construct. Intron 1 is 540 bp and initially we tested two constructs with non-overlapping 205 bp deletions (intron1m3 and intron1m4, Fig 6). These deletions did not include the first 50 or last 80 nucleotides of the intron so as not to disrupt splicing, which was verified by RT-PCR for all constructs. Nucleotides 51–255 of intron 1 (including the SURE-1 element) were deleted in intron1m3 and nucleotides 256–460 were deleted in intron1m4 (Fig 6). The intron1m3 construct retained sucrose-induced GUS activity and expression, while no GUS expression or activity was observed in sucrose-treated seedlings of intron1m4 (Fig 6). These results further verify that the SURE-1 element is not important for the sucrose-response of *PAP1* because removal of the SURE-1 element (in intron1m3) did not affect the sucrose-response.

Smaller (40–60 bp) deletions were made within the 205 bp region deleted in intron1m4 and these constructs were tested for sucrose responsiveness (intron1m5- 8, Fig 6). In addition, two potential cis-elements within this 205 bp region were also mutagenized (intron1m9-11, Fig 6). These potential cis-elements included a 10 nt palindromic G-box sequence with similarity to an abscisic acid response element (G/ABRE consensus: (T/C)ACGTG(T/G)C, *PAP1* sequence: AACACGTGTT) [38] and a sequence resembling Coupling Element 3 (ACGCGTGTCCTC) that has been found to be associated with ABREs (PAP1 sequence: ACGCTTGTCTTC) [39, 40] (Fig 6). Both of these elements were mutated individually (intron1m9 and 10) as well as together (intron1m11). All three of these constructs retained a sucrose-response, indicating these putative elements are not important for sucrose-dependent expression (Fig 6).

Deletion constructs intron1m5 and 6 also retained sucrose-responsiveness, but sucrose-induced GUS activity was absent in constructs intron1m7 and intron1m8. RT-PCR and sequencing confirmed that splicing was not affected in these mutants. This identified a 90 bp region of intron 1, that when deleted (in constructs intron1m7&8), abolished the sucrose-response.

### A 90 bp region is sufficient for conferring a sucrose response

**Fig 7:**
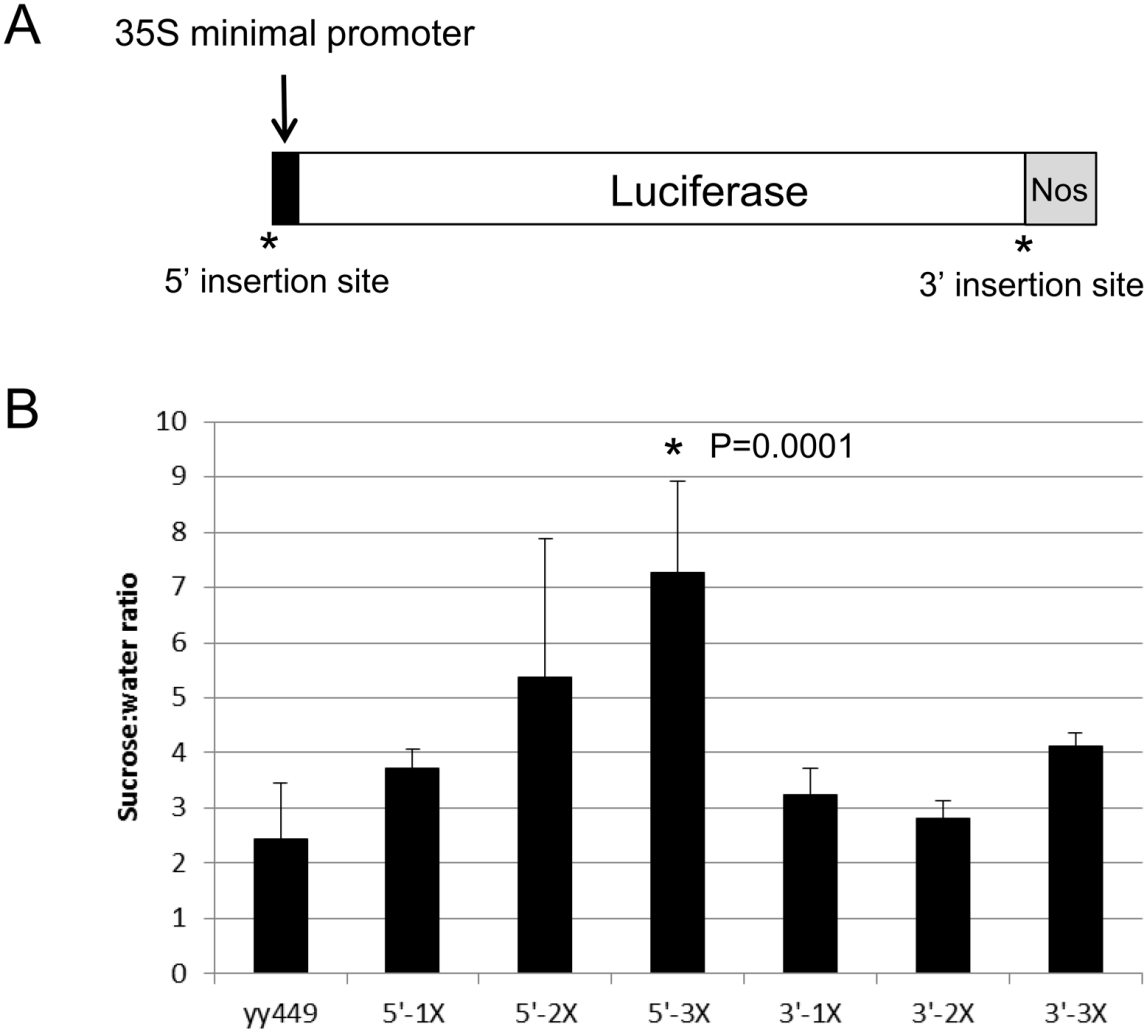
Sucrose-responsiveness of 90 bp sucrose response element: minimal promoter constructs. A. One to 3 copies of the 90 bp SRE was inserted upstream (5’) of the 35S minimal promoter or downstream of the *LUC* coding sequence (3’) in the vector yy449. B. Sucrose response of transgenic seedlings harboring the empty vector (yy449) or 1–3 copies of the 90 bp SRE in either the 5’ or 3’ insertion site. Error bars represent standard deviation of 2–4 independent single-copy homozygous transgenic lines. Significance was calculated using ANOVA to examine the influence of construct on the sucrose to water ratio. After determining there was a significant influence of construct, a post-hoc Tukey’s HSD was performed using a confidence level of 0.05.

To determine if this 90 bp region in the intron of *PAP1* is sufficient to confer a sucrose response, we cloned 1–3 copies of this region upstream of the 35S minimal promoter and *LUC* reporter gene (5’-1X, 5’-2X, 5’-3X; Fig 7). We also cloned the same fragments downstream of *LUC* (3’-1X, 3’-2X, 3’-3X; Fig 7) to test whether the spatial context of the element was important. Relative to the empty yy449 vector, only the 5’-3X construct had a significantly higher sucrose response although there was a trend of increased sucrose response as the number of 90 bp elements increased. Constructs with the 90 bp element 3’ of *LUC* did not show a significant sucrose response.

## Discussion

### Intronic sequences are important in regulating the sucrose responsiveness of *PAP1*

Given the fundamental roles that sugars play in plant metabolism, understanding how sugars also serve as signaling molecules is vital towards gaining a comprehensive understanding of plant growth and development. Sugars such as glucose and sucrose are known to regulate the expression of large numbers of genes as evidenced by reporter gene studies [11, 14–16, 18, 19, 21, 22, 41–44] and large scale gene expression analyses [4, 45–50]. We used reporter gene assays in combination with mutant approaches to identify sequences important for regulating the sucrose response of *PAP1*. Two notable findings have come from this work. First, the sequence shown to be necessary and sufficient for the sucrose response of *PAP1* was located in an intron. Although there are many genes that are known to be regulated by intronic sequences, including the floral *Arabidopsis* MADS box gene *AGAMOUS* [51, 52], and several sucrose transporters including *AtSUC1*, *AtSUC9*, and *LeSUT1* [34, 53], there are no reports of sugar response elements in an intron. Furthermore, bioinformatic approaches to identify cis elements typically limit searches to upstream regions [54, 55]. Second, despite the presence of 3 known SUREs in the promoter and first and second introns of *PAP1*, none were found to be important. The promoter of *PAP1*, which contains a SURE-2 element with extended identity to the SURE-2 element of patatin was not sufficient for conferring sucrose responsiveness. And removal of the SURE-2 in intron 2 (by deletion of the entire intron) did not abolish the sucrose response of *PAP1*. In addition, mutation of SURE-1 did not abolish the sucrose response. Although it is possible that our two mutations of SURE-1 failed to alter the element sufficiently for complete loss of function, complete removal of SURE-1 (in construct intron1m3) does not affect the sucrose response thus demonstrating this element is dispensable for sucrose regulation. Although SURE-1 has been implicated in regulating sucrose responsiveness in numerous genes [19, 32, 43, 44, 56–59], in at least one other case SURE elements were not important [60]. Furthermore, the presence of SURE elements in promoter sequences of *Arabidopsis* genes was not or only weakly correlated with the regulation of these genes by sucrose [54].

### A 90 bp intronic sequence functions as a sucrose response element

In addition to SURE-1 and SURE-2, other SREs that have been identified include the B-box [32, 61], SP8 elements [33], TGGACGG element [17], ATCATT element [62], site II elements (TGGGCY) [62], SUC-6 element (GAANGAGANGA) [54], CMSRE-1 and -2 [20], as well as several elements that respond to both sucrose and reactive oxygen species [54]. None of these motifs are present within the 90 bp region we identified, although this sequence shares similarity to elements such as SURE-1, SURE-2, SP8, B-box site M1, and TTACTA in being AT-rich (67% AT content for 90 bp region).

When placed outside the context of an intron upstream of the 35S minimal promoter, the 90 bp sequence was able to function as a SRE, although it only had a robust sucrose response when present in 3 tandem copies. The sucrose response of constructs with one copy of the 90 bp region was not significantly different from the empty vector unlike what was observed in *PAP1wg* lines, which contain one copy of the 90 bp region in the context of intron 1. This indicates that other sequences may be required for a robust sucrose-response and/or that the spatial context of the 90 bp sequence is important. Evidence for the latter is suggested by the fact that the 90 bp sequence did not function when placed downstream of *LUC* (in 3’ constructs). In these constructs the element was located 1700 bp downstream of the start codon, while in the context of the *PAP1* gene the element is 491 bp downstream of the start codon. The difference in distance of the element relative to the transcriptional start may be one reason that the element did not function effectively.

### Contribution of sucrose and other regulatory elements in the expression of *PAP1*

The correlation between sucrose responsiveness and pattern of GUS staining in our transgenic plants suggests that sequences conferring sucrose responsiveness are also responsible for the observed expression pattern of *PAP1* in plants. Removal of intron 1 (in constructs *PAP1wgΔInt1*:*GUS* and *PAP1wgΔInts*:*GUS*) severely limits GUS expression quantitatively and qualitatively as compared to *PAP1wg*:*GUS* lines. Furthermore, *PAP1wgΔInt2*:*GUS* and both SURE-1 mutants (intron1m1 and intron1m2) retained sucrose responsiveness and both had GUS staining patterns nearly identical to *PAP1wg*:*GUS* lines. The ability of sucrose to govern the expression of *PAP1* may be related to the protective role of anthocyanins during stress responses. For instance, stress conditions such as high light and cold are known to increase sucrose content and accumulation of anthocyanins in these conditions may serve to protect cells from damage by virtue of their ability to absorb light and serve as antioxidants and osmoprotectants [63–66].

Differences in the expression pattern between *PAP1pro*:*GUS* and *PAP1wg*:*GUS* lines further underscores the complexity of *PAP1* regulation. *PAP1wg*:*GUS* plants showed GUS staining that was reminiscent of anthocyanin pigmentation in seedlings and plants (hypocotyls in young seedlings and leaves with noticeable staining along major veins) and in several other tissues as well (cauline leaves, inflorescences, immature and mature flowers, siliques). RT-PCR confirmed that *PAP1* transcript was present in all of these organs and tissues in wild-type *Col-0* plants. The pattern of GUS staining in *PAP1pro*:*GUS* was remarkably different and nearly opposite that of the *PAP1wg*:*GUS* plants. We first observed staining in the roots of 20 day old *PAP1pro*:*GUS plants*, but this was at a different location than the root staining observed in *PAP1wg*:*GUS* plants. No rosette staining was observed in *PAP1pro*:*GUS* lines while strong rosette expression was observed in *PAP1wg*:*GUS* lines. Both lines had GUS expression in mature flowers but *PAP1pro*:*GUS* lines had strong petal and no sepal staining while *PAP1wg*:*GUS* lines had strong sepal and no petal staining. Because the *PAP1* promoter sequence is present in the *PAP1wg*:*GUS* construct, the discrepancy in staining patterns may be explained by the presence of sequences within the introns and/or exons of *PAP1* that repress petal staining. Repressor elements are most likely within the exons of *PAP1* because petal staining is not observed in the *PAP1wgΔInts*:*GUS* line, which contains the *PAP1* promoter plus exons, but no introns. Furthermore, because the *PAP1wgΔInts*:*GUS* line had very limited GUS staining that was nothing like the staining observed in *PAP1pro*:*GUS lines*, *PAP1* exons must repress all promoter expression elements and contain sequences conferring the weak expression observed in rosette and cauline leaves.

## Conclusions

A 90 bp intronic sequence was identified as important for regulating the sucrose response of *PAP1* and was sufficient for conferring a sucrose response to a reporter gene when present in multiple copies. Intronic sequence was also shown to be important for the proper pattern of *PAP1* gene expression. Pigmentation of flowers, fruits, and vegetables remains a target for crop improvement [67] and understanding the many layers of *PAP1* regulation may lead to new approaches for modifying anthocyanin content of these plants.

## Materials and Methods

### Sucrose treatment

Surface-sterilized seeds were put into a 250 ml flask containing 50 ml of sterilized ½-strength MS basal salts pH 5.7 (Phytotechnology Laboratories, Shawnee Mission, KS). Seeds were kept at 4°C for 2 days then transferred to a continuous light or dark chamber with shaking at ~100 rpm. Four days after transfer to the chamber, sucrose (or an equivalent volume of water) was added to 90 mM from a 1.8 M stock. Seedlings were harvested at the indicated timepoints following addition of either sucrose or water.

### RT-PCR

Seedlings were harvested and frozen immediately in liquid N_2_. Total RNA was extracted using the RNeasy Plant mini kit (Qiagen, Valencia, CA) and treated with DNase I (Fermentas, Glen Burnie, MD). Two μg of RNA was reverse transcribed using MMLV Reverse Transcriptase (Ambion, Austin, TX) according to the manufacturer’s protocol. Two to 5 ul of cDNA were used in 20 μl PCR reactions. *PAP1* and actin were amplified using primers from Teng et al (MYB75F/R and ACT8F/R) [12]. *GUS* transcript was amplified using the following primers: GusF: 5’-ACCGTTTGTGTGAACAACGA-3’ and GusR: 5’-GGCACAGCACATCAAAGAGA-3’. In some experiments *PAP1*:*GUS* chimera transcript was amplified using the following primers: PAP1_2031F: 5’-CGAAAAGGTGCTTGGACTACT-3’ and GUS_424R: 5’-TCTGCCAGTTCAGTTCGTTG-3’.

### RT-qPCR

Total RNA was extracted using the RNeasy Plant mini kit (Qiagen, Valencia, CA) and treated with TURBO DNA-free kit (Ambion, Austin, TX). One μg of RNA was reverse transcribed using iScript Reverse Transcription Supermix (BioRad, Hercules, CA) according to the manufacturer’s protocol. Twenty μl reactions were prepared using 1 ul of cDNA, 0.5 to 0.8 uM primer, and SYBR Premix Ex Taq II (Perfect Real Time) reagent (Takara Bio Inc, Otsu, Shiga Japan). All reactions were performed in triplicate. *PAP1* and ubiquitin primer sequences are from Solfanelli et al [13]. Amplification was carried out on a Roche 480 Light Cycler (Roche, Madison, WI) with the following cycling parameters: One cycle of 95°C for 5 min followed by 45 cycles of amplification (95°C for 10 s: 60°C for 10 s; 72°C for 10 s). Cq values were calculated using the Light Cycler 480 SW 1.5 software and the Absolute quantification/2^nd^ derivative method. Primer efficiencies were calculated from the slope of Cq values plotted as a concentration of cDNA (ranging from 0.01–1 μg) and were 102 and104% for *PAP1* and ubiquitin, respectively. Cq values and primer efficiencies were input into the Relative Expression Software tool (REST) 2009 version [68] and fold changes were determined relative to ubiquitin.

### Anthocyanin quantification

Anthocyanin quantification was modified from Solfanelli et al [13]. Anthocyanins were extracted in 1 ml 1% HCl in MeOH for 16–24 hours at 4°C. 0.9 ml of the extract was combined with 0.9 ml water and 1 ml chloroform. Extracts were vortexed and clarified by low speed centrifugation. 0.9 ml of the aqueous phase was removed and Abs 530 and 657 were determined. Anthocyanin content is reported as Abs 530-657/mg fresh weight.

### PAP1 promoter, whole gene, and delta intron-reporter constructs

The 2 kb 5’-upstream region of the *PAP1* gene (At1g56650) was amplified from *A*. *thaliana* genomic DNA (*Col-0*) using the Expand High Fidelity PCR system (Roche Applied Science, Indianapolis, IN) and the following primers, which contain restriction enzyme sites (underlined): PAP1HindIIIF: 5’-AACACTACAAAAAAGCTTAACTGCATTTAG-3’ and PAP1proSmaIR: 5’-TGGACGAACCCTCCCGGGAACAAAGATAG-3’ or PAP1HindIIIF and PAP1HindIIIR: 5’-TGGACGAACCCTAAGCTTAACAAAGATAG-3’. A 3379 bp fragment containing the 2 kb 5’-upstream region and all exons and introns of *PAP1* gene was amplified using PAP1HindIIIF and PAP1wgSmaIR: 5’-CAAATGTTCGAACCCGGGATCAAATTTCAC-3’ or PAP1wgHindIIIR: 5’-CAAATGTTCGAAAAAGCTTTCAAATTTCAC-3’. The whole gene constructs were designed to be translationally fused to either the *GUS* or *LUC* ORF and the insertion of a HindIII site for cloning the whole gene construct into pLPTV-BAR resulted in a change of the last amino acid of PAP1 from Asp to Glu.

PCR products were cloned into the pDrive vector using the Qiagen PCR Cloning Kit (Qiagen, Valencia, CA) and sequenced. pDrive containing the 2 kb *PAP1* promoter amplified with PAP1HindIIIF and PAP1proSmaIR was first digested with Sma I and the resulting 3676 bp fragment (containing the 2 kb *PAP1* promoter plus vector sequence) was isolated. This fragment was partially digested with Hind III and a 2005 bp fragment was isolated and cloned into the Sma I and Hind III sites of pBI101.3. pDrive containing the 2 kb PAP1 promoter amplified with PAP1HindIIIF and PAP1proHindIIIR was partially digested with Hind III and the resulting 2005 kb fragment was isolated and cloned into the Hind III site of the pLPTV-BAR vector. pDrive containing the whole gene *PAP1* sequence amplified with PAP1HindIIIF and PAP1wgSmaIR was first digested with Sma I and the resulting 5543 bp fragment was isolated. This fragment was partially digested with Hind III and a 3379 bp fragment was isolated and cloned into the Sma I and Hind III sites of pBI101.3. pDrive containing the whole gene *PAP1* promoter sequence amplified with PAP1HindIIIF and PAP1wgHindIIIR was partially digested with Hind III and the resulting 3379 kb fragment was isolated and cloned into the Hind III site of the pLPTV-BAR vector. Sequence identity and orientation were confirmed by sequencing.

To generate *PAP1wg* constructs lacking one or both introns, a full length *PAP1* cDNA was amplified from *Arabidopsis* seedlings. To create *PAP1wgΔIntron2*, a 531 bp Age I-Spe I fragment (containing Intron 2) was removed and replaced with a 442 bp Age I-Spe I fragment from *PAPI* cDNA. To create *PAP1wgΔIntron1*, a synthetic DNA fragment was constructed from the Sca I site in the 5’-upstream region of the *PAP1* gene to the Age I site in Exon 2 and omitted Exon 1 (GENEART, Regensburg, Germany). This 430 bp Sca I- Age I fragment was used to replace the corresponding 969 bp fragment from *PAP1wg* and *PAP1wgΔIntron2* to create *PAP1wgΔIntron1* and *PAP1wgΔIntrons1&2*, respectively.

### Intron1m1-8 reporter constructs

Mutations were introduced by PCR [69]. For the intron1m1 mutation, a 50 μl reaction was performed with 50 ng pBI101.3-PAP1wg plasmid, 200 μM dNTPs, 6% DMSO, 2.5 U PFU Ultra HF polymerase (Stratagene, La Jolla, CA) and 150 ng of the following primers: mut1F: 5’-TAATCACTACCAATAGTCTTCGTTCTCTCTATTTGAGATTCAGAAAATTGATTAATACCCGG-3’

mut1R: 5’-CCGGGTATTAATCAATTTTCTGAATCTCAAATAGAGAGAACGAAGACTATTGGTAGTGATTA-3’. Cycling conditions were 95°C for 1 minute followed by 16 cycles of 95°C for 50s, 60°C for 1 min 68°C for 32 min, and a final extension time of 68°C for 7 min. Reactions were digested with 20 U of DpnI (New England Biolabs, Ipswich, MA) for 2 hrs and 5 μl was used to transform DH5α cells. For the intron1m2 mutation, a 50 μl reaction was performed with 50 ng pDrive-PAP1wg plasmid and the following primers according to Zheng *et al*: mut2F: 5’- gtcttcgttctctctaaatactaatcagaaaattgattaatacccggtattaaaaaaaaaaaaaaaaatttgtttaaatgagtac-3’ and mut2R: 5’- ttaatcaattttctgattagtatttagagagaacgaagactattggtagtgattatagatattcatatttgtgtgtgtgt-3’ [70]. Plasmids with the intron1m2 mutation were digested with Sca I and Age I and the resulting 970 bp fragment (containing intron 1) was used to replace the corresponding fragment in pBI101.3-PAP1wg.

For the intron1m3 and intron1m4 deletion mutations, single primers were used to generate mutations in pDrive-PAP1wg according to Makarova *et al* [71]. The primers used were: mut3R: 5’- GTAAAAATCTTCGTTTTTTGTGTGTGTGTGTGTCGGTTAGTGTGT-3’ and mut4F: 5’-TTTCTTTTGCTGTTCGTATTTGTTTTACACCTATAAAATATATAGAAGGAG-3’. Mutations were incorporated into pBI101.3-PAP1wg as described above.

For the intron1m5-8 deletions, synthetic DNA fragments were constructed in the context of the Sca I-Age I fragment that encompasses intron 1, and incorporating the specific deletions shown in Fig 5 (GENEART, Regensburg, Germany). These Sca I- Age I fragments were used to replace the corresponding 969 bp fragment from PAP1wg.

### Minimal promoter constructs

For the 5’ constructs, a synthetic DNA fragment was constructed with the 90 bp region of interest flanked by HindIII and BamHI (5’) and BglII (3’) restriction sites (GeneArt^®^ Gene Synthesis by Life Technologies): AAGCTTAAAGGGATCCtaaatgaattcgtgggaaaattttgtatgaacacgtgtttctgtgttggaacagttctttatttttattggtgtgcatagattcttcctgAGATCT. This fragment was digested with HindIII and BglII and ligated into HindIII and BamHI-digested yy449 vector (Genbank accession AB638628.1). This process was repeated once or twice more to obtain plasmids with two (192 bp) or 3 (288 bp) copies of the 90 bp region. For the 3’ constructs, 1–3 copies of the 90 bp fragment were synthesized with flanking XbaI sites. Multiple copy elements were separated with the spacer AGATCC. These fragments were cloned into the XbaI site of yy449.

### Generation of transgenic plants

Plasmids were transformed into *Agrobacterium tumefaciens* strain GV3101 by electroporation. *A*. *thaliana* ecotype *Col-0* was transformed by the floral dip method as described [72]. Seeds were surface-sterilized by treating with 95% ethanol for 10 min followed by 20% bleach supplemented with 0.1% Tween-20 for 5 min, and rinsed several times in sterile water. Seeds were suspended in 0.1% agargel and plated on MS plates supplemented with 50 μg ml^-1^ kanamycin (pBI101.3) or sowed directly on soil and sprayed with Liberty herbicide (pLPTV). Resistant plants were checked by PCR for the presence of the transgene.

### Gus staining

Seedlings were stained in 100 mM sodium phosphate buffer, pH 7, 10 mM EDTA, 0.1% Triton X-100, 2 mM K_4_Fe(CN)_6_, 2 mM K_3_Fe(CN)_6_, and 1 mg ml^-1^ 5-bromo-4-chloro-3-indolyl ß-D glucuronide for 24–48 hours at 37°C. Seedlings were then repeatedly destained using 70% ethanol and photographed.

### Luciferase assay

Seedlings ground to a fine powder and luciferase activity was measured using the Luciferase Assay System (Promega, Madison, WI) according to the manufacturer’s protocol. Protein content in cell extracts was measured by Bradford assay using BSA as standard.

## Supporting Information

S1 Fig GUS staining of 3, 5, and 7 d old *PAP1* promoter and whole gene lines. (PPTX)

S2 Fig GUS staining of 14 d old *PAP1* promoter and whole gene lines. (PPTX)

S3 Fig GUS staining and RT-PCR of 20 d old *PAP1* promoter and whole gene lines. (PPTX)

S4 Fig GUS staining in *PAP1wgΔInt1*:*GUS* and *PAP1wgΔInts*:*GUS* lines. (PPT)
