# Supplementary figures and images for: Intronic Sequence Regulates Sugar-Dependent Expression of Arabidopsis thaliana Production of Anthocyanin Pigment-1/MYB75
Source: PLoS One. 2016 Jun 1;11(6):e0156673. doi: 10.1371/journal.pone.0156673 (PMC4889055; doi:10.1371/journal.pone.0156673)

## Slide 1
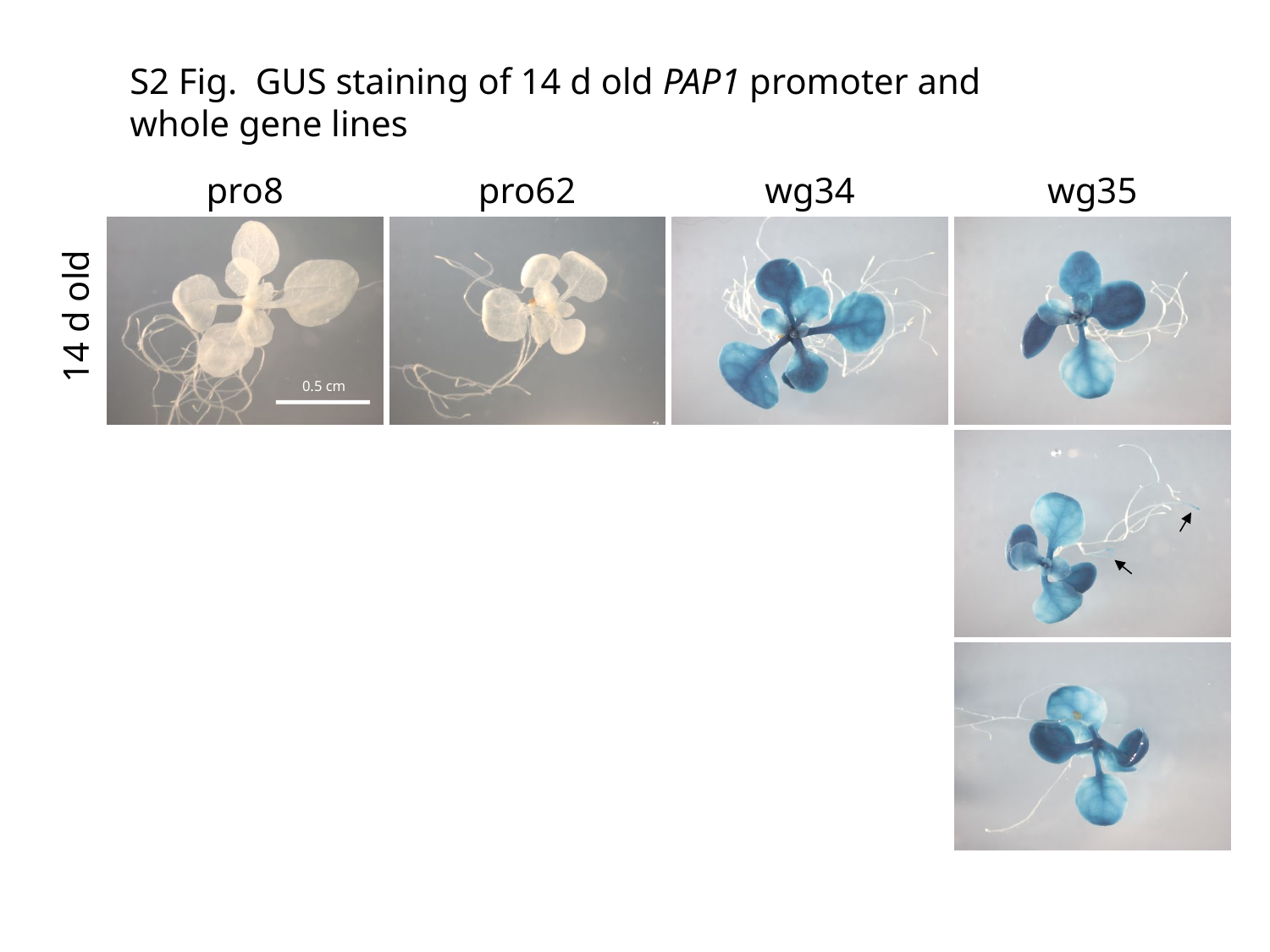

S2 Fig. GUS staining of 14 d old PAP1 promoter and whole gene lines
pro8
pro62
wg34
wg35
14 d old
0.5 cm

Supplement: S2 Fig — (PPTX) [file pone.0156673.s002.pptx]
