# Supplementary material for: Intronic Sequence Regulates Sugar-Dependent Expression of Arabidopsis thaliana Production of Anthocyanin Pigment-1/MYB75
Source: PLoS One. 2016 Jun 1;11(6):e0156673. doi: 10.1371/journal.pone.0156673 (PMC4889055; doi:10.1371/journal.pone.0156673)
Supplement: S3 Fig — (PPTX) [file pone.0156673.s003.pptx]

## Slide 1
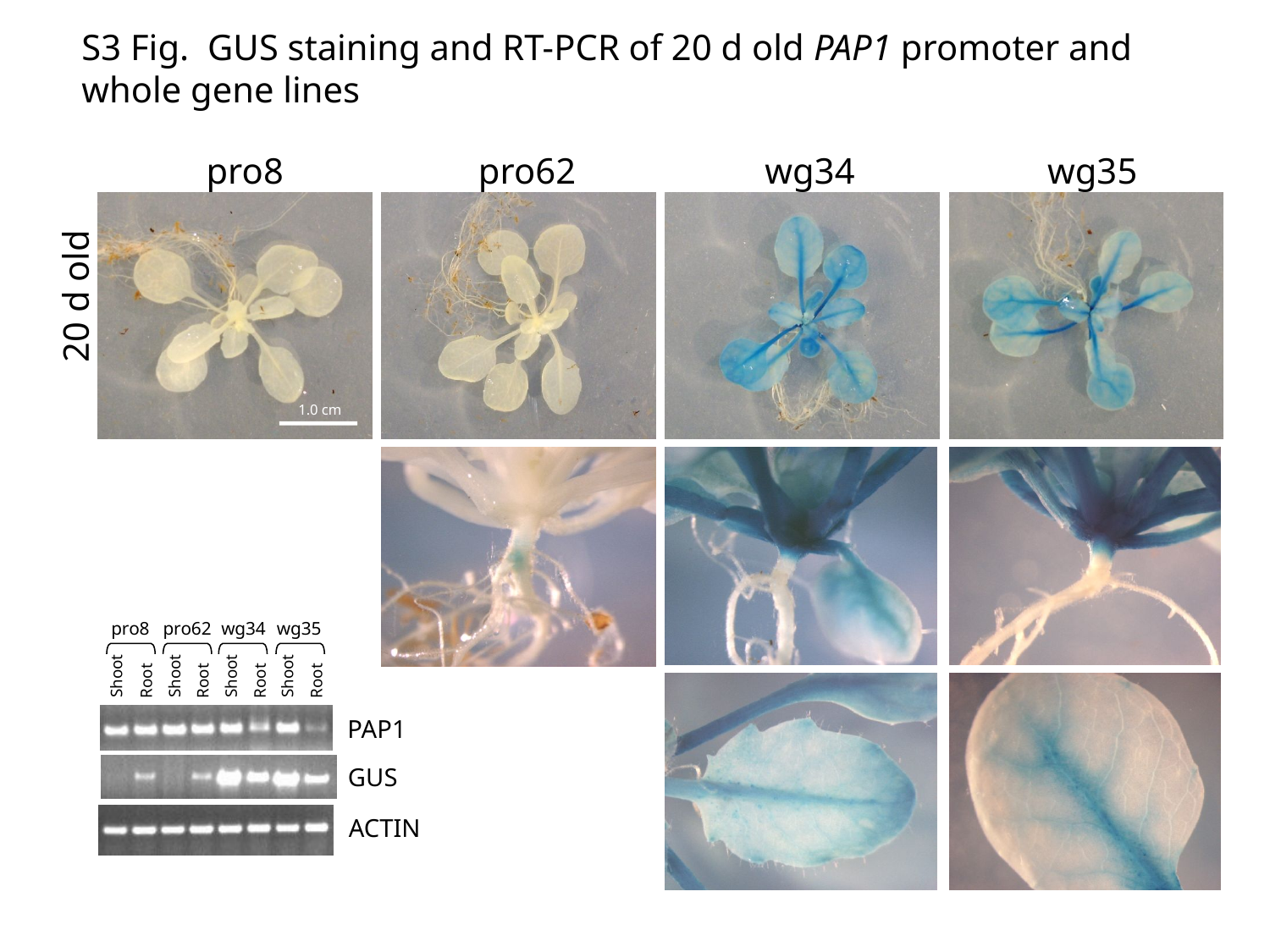

S3 Fig. GUS staining and RT-PCR of 20 d old PAP1 promoter and whole gene lines
pro8
pro62
wg34
wg35
20 d old
1.0 cm
pro8
pro62
wg34
wg35
Shoot
Shoot
Shoot
Shoot
Root
Root
Root
Root
PAP1
GUS
ACTIN
