# Supplementary material for: Intronic Sequence Regulates Sugar-Dependent Expression of Arabidopsis thaliana Production of Anthocyanin Pigment-1/MYB75
Source: PLoS One. 2016 Jun 1;11(6):e0156673. doi: 10.1371/journal.pone.0156673 (PMC4889055; doi:10.1371/journal.pone.0156673)
Supplement: S4 Fig — (PPT) [file pone.0156673.s004.ppt]

## Slide 1
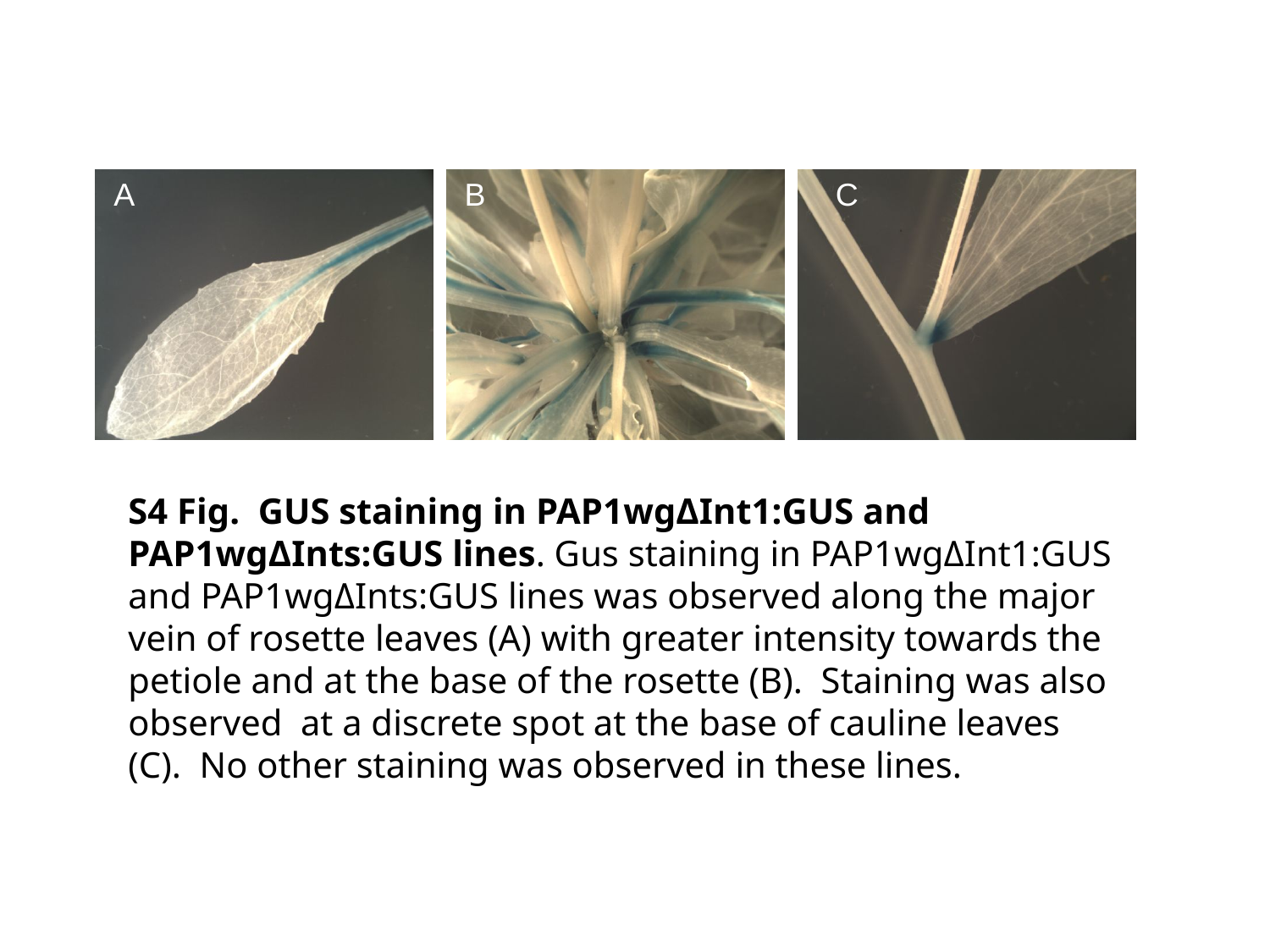

A
B
C
S4 Fig. GUS staining in PAP1wgΔInt1:GUS and PAP1wgΔInts:GUS lines. Gus staining in PAP1wgΔInt1:GUS and PAP1wgΔInts:GUS lines was observed along the major vein of rosette leaves (A) with greater intensity towards the petiole and at the base of the rosette (B). Staining was also observed at a discrete spot at the base of cauline leaves (C). No other staining was observed in these lines.
